# Supplementary material for: Equine Management in UK Livery Yards during the COVID-19 Pandemic—“As Long As the Horses Are Happy, We Can Work Out the Rest Later”
Source: Animals (Basel). 2021 May 14;11(5):1416. doi: 10.3390/ani11051416 (PMC8156832; doi:10.3390/ani11051416)
Supplement: Supplementary file 1 [file animals-11-01416-s001.zip › animals-1180994-supplementary.pdf]

animals

MDPI

Article

Equine Management in UK Livery Yards during the COVID-19 Pandemic—"As Long As the Horses Are Happy, We Can Work Out the Rest Later"

Tamzin Furtado [1,*], Elizabeth Perkins [2], Catherine McGowan [1] and Gina Pinchbeck [1]

1 Institute of Infection, Veterinary and Ecological Sciences, University of Liverpool, Cheshire CH64 7TE, UK; tfurtado@liverpool.ac.uk (T.F.); cmcgowan@liverpool.ac.uk (C.M.); ginap@liverpool.ac.uk (G.P.)
2 Institute of Population Health, University of Liverpool, Liverpool L69 3GL, UK; lizp@liverpool.ac.uk (E.P.)
* Correspondence: tfurtado@liverpool.ac.uk

Citation: Furtado, T.; Perkins, E.; McGowan, C.; Pinchbeck, G. Equine Management in UK Livery Yards during the COVID-19 Pandemic—"As Long As the Horses Are Happy, We Can Work out the Rest Later". *Animals* **2021**, *11*, 1416. https://doi.org/10.3390/ani11051416

Academic Editor: Hayley Randle, Jane Williams, Mandy Paterson

Received: 28 March 2021
Accepted: 13 May 2021
Published: 14 May 2021

Publisher's Note: MDPI stays neutral with regard to jurisdictional claims in published maps and institutional affiliations.

Copyright: © 2021 by the authors. Licensee MDPI, Basel, Switzerland. This article is an open access article distributed under the terms and conditions of the Creative Commons Attribution (CC BY) license (http://creativecommons.org/licenses/by/4.0/).

Decision making and changing equine management practices at UK livery yards as a result of COVID-19

Interview guide

These will be semi-structured interviews, loosely structured around the questions below. Based on experience, we expect that respondents are likely to talk at length, and because so little is known about this subject, the interview guide will be developed and altered as interviews progress, based on the knowledge gained from previous interviews.

Thank you for agreeing to take part in this study. You are free to talk as little or as much as you like about each of the points we will discuss, and to stop at any time if you decide you no longer want to take part. First I will ask you about the physical practicalities of the yard, like how many horses and how you keep them, before we talk about the way you manage the clients, and then how things have changed since Covid-19.

1. First, could you tell me about the yard itself:
    - How many horses/how many owners
    - DIY/Full/Part
    - How much land
    - How many stables
    - Facilities (arena etc)
    - BHS approved?
    - Staff?
    - Are you an owner or manager? How long have you been in this role?
    - Contract in place with others? Pricing?
    - What are the best things about the yard, and the less-good things?
2. Could you tell me about a typical daily routine on the yard?
3. Could you tell me about the rules and guidelines which were in place for your clients, before the covid-19 outbreak? [prompts: how were these set up? Do they change? Are they formal like in a contract? Are there any informal rules "common sense"? what happens if people break them?]
4. Sometimes clients might request something specific for their horse, such as to move stable or set up a track in their field. Have you had anything like this happen in the last year or so (prior to covid)? How did you manage the situation?

5. Do you have an idea of what you'd consider a "good client" to be, compared to a "difficult client"?
6. Thinking about the horses on your yard before Covid-19, what aspects do you think are example of good welfare? Are there things about them that you think you'd have liked to improve? [prompts: how do you decide how to manage their welfare? Have there been times you'd have to prioritise other things such as a client's wishes, over the welfare of the horse? What is good welfare to you/what does a "happy horse" look like?]
7. Now I'd like to ask about your experience since the start of the Covid-19 pandemic.
   - Have you put in place any changes to the rules and regulations for clients since the outbreak?
   - Have you put in place any changes to the horses' management?
   - Changed to the environment?
   - How have these changes been received by clients?
   - Have you had to put in place any extra or special rules because of specifically vulnerable people (e.g. clients or in your own family perhaps), that are different to what you might have done if there was nobody who was especially vulnerable?
   - Do you think that those changes have impacted the horse's welfare? [In what ways? Which changes have improved their welfare? Which changes have reduced their welfare? Do you think you'd like to continue some of those changes longer term? Anything you would absolutely not do again?]
   - Have those changes impacted other things, like your clients' needs, your land management, your staff, or your business model?
   - How do you see things progressing in the current weeks and months?
   - If you could go back in time to the start of the outbreak, knowing what you know now, what would you have done differently on your yard?

Debriefing:

Thank you for taking part in the study, we really appreciate your time. Your interview will be transcribed, then anonymised and placed with the other participants' data, so that we can look for strategies that might help other livery yard owners and managers in future. If you have any questions at all please feel free to contact me at any time.

Would you be interested in being involved in future interviews, to see how things are going in the next 3-6 months?
